# Health disparities and underserved populations: a potential solution, medical school partnerships with free clinics to improve curriculum

**DOI:** 10.3402/meo.v20.27535

**Published:** 2015-04-21

**Authors:** Lynn M. VanderWielen, Allison A. Vanderbilt, Steven H. Crossman, Sallie D. Mayer, Alexander S. Enurah, Samuel S. Gordon, Melissa K. Bradner

**Affiliations:** 1 Department of Family Medicine, School of Medicine, University of Colorado Denver, Aurora, CO, USA; 2 Center on Health Disparities, Virginia Commonwealth University, Richmond, VA, USA; 3 School of Medicine, Virginia Commonwealth University, Richmond, VA, USA; 4 Department of Family Medicine and Population Health, School of Medicine, Virginia Commonwealth University, Richmond, VA, USA; 5 Department of Pharmacotherapy and Outcomes Science, School of Pharmacy, Virginia Commonwealth University, Richmond, VA, USA; 6 Division of Internal Medicine, School of Medicine, University of Colorado Denver, Aurora, CO, USA; 7 School of Medicine, University of Colorado Denver, Aurora, CO, USA

**Keywords:** health disparities, uninsured, medical education, underserved populations, free clinics

## Abstract

Health-care educators share the social responsibility to teach medical students about social determinants of health and health-care disparities and subsequently to encourage medical students to pursue residencies in primary care and medical practice in underserved communities. Free clinics provide care to underserved communities, yet collaborative partnerships with such organizations remain largely untapped by medical schools. Free clinics and medical schools in 10 US states demonstrate that such partnerships are geographically feasible and have the potential to mutually benefit both organizational types. As supported by prior research, students exposed to underserved populations may be more likely to pursue primary care fields and practice in underserved communities, improving health-care infrastructure.

The health-care system in the United States is burdened with disparities associated with a panoply of factors including complex interactions between race/ethnicity and socioeconomic status (1), geographic access to care (2), and health insurance status (3–7). For instance, insurance status is one of the strongest predictors of cancer screening utilization (3–5) and advanced disease progression upon treatment (6), and the association between race/ethnicity and infant mortality rates is widely documented in the academic literature (8, 9). Additionally, individuals living in health professional shortage areas are less likely to receive medications for cardiovascular disease prevention, including statins and warfarin, especially when uninsured (2).

Over 57 million individuals live in 5,864 designated primary care shortage areas in the United States (10). By definition, individuals in these urban and rural communities face a deficit of primary care providers in four primary care specialties: general or family practice, general internal medicine, pediatrics, and obstetrics and gynecology (11). Although primary care physicians compose only 37% of the physician workforce, they provide 56% of all physician office visits (12). Experts argue that the United States will face a serious shortage of primary care physicians in the near future (12), likely reducing further the access to primary care services for medically underserved individuals.

Health-care professional students who are exposed to underserved populations during education and training are more likely to care for this same population once in practice (13); this may strengthen the health-care infrastructure in underserved communities (14). In fact, primary care physicians who complete residency training in community health centers (safety-net providers for the uninsured and other vulnerable populations) are significantly more likely to practice in medically underserved areas (15). Finally, medical students who train with underserved populations are thought to learn and rediscover social responsibility and further understand the social determinants of health (16).

To encourage students to pursue primary care fields, medical schools and students across the country have embraced training opportunities in underserved areas. For instance, through the group Primary Care Progress, hundreds of students and faculty at the University of Colorado Anschutz Medical Campus have been working with the Aurora community to create an interdisciplinary student-run free clinic to meet the needs of the underserved. The DAWN clinic (Dedicated to Aurora’s Wellness and Needs) does provide integrated primary care and serve as a patient-centered medical home for the uninsured population of Aurora, while offering an opportunity for health students to learn and collaborate in an interprofessional setting (17). The International/Inner City/Rural Preceptorship program at Virginia Commonwealth University School of Medicine partners with a local free clinic to provide enhanced teaching practice experience for program participants (18). This unique primary care learning experience partners medical students, pharmacy students, interpreters, and a variety of levels of learners to care for diverse patients within two free clinics, with a goal of accommodating the working uninsured. Students are precepted closely by health-care professional faculty, are supported by free clinic staff, and are challenged to work effectively on a team while addressing access, socioeconomic, language, and educational barriers.

Free clinics, however, remain an underutilized academic institution partnership. The United States is home to over 1,000 free clinic organizations that operate in 49 states (excluding Alaska) and the District of Columbia (19) (Table 1). Free clinics are nonprofit organizations that provide medical, dental, pharmacy, and mental health services or prescriptions to mostly uninsured patients and rely heavily on volunteers to provide clinical and administrative expertise. These free clinics in the United States annually provide health care to nearly 1.8 million people in the form of over 3.5 million medical and dental visits (19). Furthermore, these organizations have the potential to offer medical students an opportunity to serve underserved populations in a supervised environment, while simultaneously supporting the mission of the free clinic, because these organizations cannot exist without the support of health-care providers and the greater health-care community (20).

**Table 1:** Data for free clinics and medical schools in Virginia, North Carolina, South Carolina, Pennsylvania, Ohio, Michigan, Texas, California, Washington, and Missouri

	*N*
Free clinics	435
Medical schools	60
Average distance from medical school to closest free clinic	9.2 miles (SD=13.3 miles)

The fate of free clinics following full implementation of the Patient Protection and Affordable Care Act (ACA) remains to be seen, although scholars have argued their necessity will remain unchanged (20). Despite the positive impact that the ACA will have on reducing the number of the uninsured, it is estimated that approximately 20 million people will remain without insurance (21). It is anticipated that the residual uninsured population will include a larger proportion of undocumented individuals (21, 22) and others who are noncompliant with the individual mandate (23). Experts agree that the remaining uninsured population is likely to continue to seek care in organizations that contribute to the health-care safety net (24). Partnerships between medical schools and free clinics may secure a role for free clinics in the health-care delivery environment as these clinics have served the underserved in the United States for decades. Therefore, the purpose of this paper is to examine the proximity of medical schools to free clinics and implications for changes in public health policy and medical education.

## Current medical schools and free clinics

To demonstrate geographic feasibility, free clinic and medical school (doctorate of medicine and osteopathic medicine) addresses from Virginia, North Carolina, South Carolina, Pennsylvania, Michigan, California, Texas, Washington, Missouri, and Ohio were geocoded and mapped utilizing ArcMAP software (25). These states were selected as a sample in order to cover all geographical regions of the United States. Medical school addresses (admissions offices) were found on medical school websites, and free clinic state association websites offered free clinic physical addresses. For the free clinics that had multiple sites, each site was individually included.

**Fig. 1:**
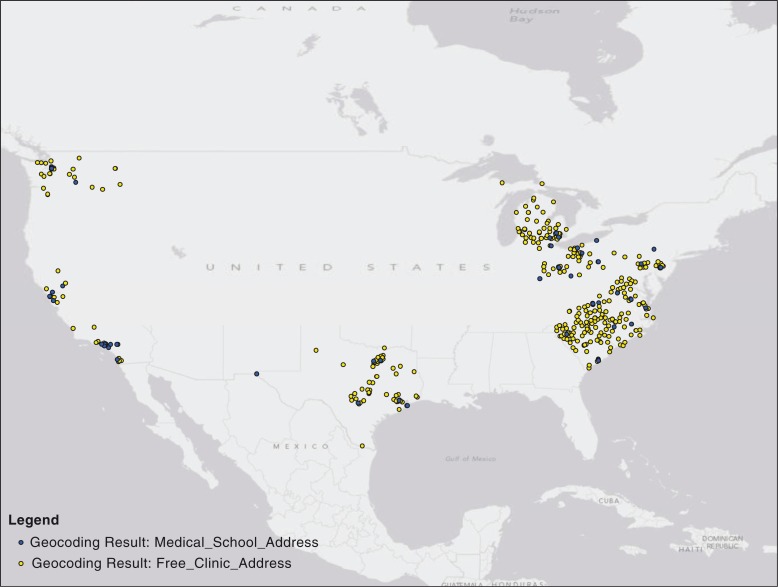
Free clinic and medical school locations throughout Virginia, North Carolina, South Carolina, Pennsylvania, Ohio, Michigan, Texas, California, Washington, and Missouri.

Four hundred and thirty-five free clinics were located in the above 10 states, along with 60 medical schools. Medical school and free clinic locations are depicted in Fig. 1. Based on this geocoded mapping, it is evident that medical schools and faculty members could support a feasible free clinic partnership to expose medical students to underserved patient populations living in their own community. Potential collaborative partnerships could include service-learning projects, integration of interprofessional education and collaboration, and public health and prevention practice, which are important for Liaison Committee for Medical Education (LCME) accreditation requirements (26) as well as for other health-care professional programs, such as Pharmacy (27). It is of pivotal importance that such partnerships support high quality care in free clinic settings with appropriate student supervision.

## Moving forward: potential positive outcomes

Although some medical institutions utilize free clinic partnerships to improve medical education, this remains an untapped resource in the United States. Medical school administration and faculty need to explore these opportunities to expose medical students to underserved populations and encourage student selection of primary care residencies and subsequent employment in underserved areas. Free clinics will benefit from the additional health professional service and support to navigate the turbulent environment of health-care reform. As health-care disparities worsen in the United States, it is the responsibility of medical school educators to maximize student awareness of the needs of the underserved and encourage student selection into primary care residencies and professional practice in underserved communities. Free clinics offer one such largely untapped, geographically feasible, and mutually beneficial opportunity to improve medical school education.

## Recommendations for curriculum

To enhance and improve the medical education curriculum, it is critical and necessary to expose medical students to free clinics, underserved populations, and health disparities in a community. With the LCME (28) requiring health disparities as part of the curriculum, it would be beneficial to both medical schools and students to increase participation with patients within the community while on clinical rotations. In addition, this opportunity can meet the needs set forth by the LCME for service learning (26) and provide care that potential patients otherwise would not receive. These initiatives can expand into rural or urban communities for medical students to experience the continuum of health care in various locations. These opportunities, not only have a potential impact for learning for students, but an impact on the curriculum and hard-to-reach communities that may be underserved or lack access to care without the support of medical students.

Health-care professionals can unite with the common curricular objectives to provide service learning opportunities to students, interprofessional education experiences, provide more options for preceptors within the community, expand clinical rotations into more diverse populations, and expose students to a variety of health disparities within the community. This situation, can be achieved across all four years of medical school and integrated throughout the medical curriculum while simultaneously meeting several LCME standards. Finally, exposing students early on to underserved communities may influence their selection with residency programs, therefore increasing the number of medical students who choose to go into family medicine or internal medicine and work at HSPAs or safety-net providers for their careers.

## Conclusion

Health-care educators, medical schools, and integrated medical curricula all share the social responsibility to teach medical students about social determinants of health and health-care disparities and subsequently encourage medical students to pursue residencies in primary care and medical practice in underserved communities. Free clinics provide care to underserved communities, yet collaborative partnerships with such organizations remain largely untapped by medical schools. Incorporating medical student experiences at free clinics into the current curriculum has the potential to influence residency selection. Students exposed to underserved populations are more likely to pursue primary care fields and consequently to contribute constructively to our national health-care infrastructure.
